# Physicochemical Characteristics of Arginine Enriched NaF Varnish: An In Vitro Study

**DOI:** 10.3390/polym12122998

**Published:** 2020-12-16

**Authors:** Mohammed Nadeem Bijle, Manikandan Ekambaram, Edward Lo, Cynthia Yiu

**Affiliations:** 1 Paediatric Dentistry, Faculty of Dentistry, The University of Hong Kong, Hong Kong; mnbijle@connect.hku.hk; 2 Paediatric Dentistry, Faculty of Dentistry, University of Otago, Dunedin 9016, New Zealand; mani.ekambaram@otago.ac.nz; 3 Dental Public Health, Faculty of Dentistry, The University of Hong Kong, Hong Kong; edward-lo@hku.hk

**Keywords:** arginine, caries, fluorides, prevention, varnish

## Abstract

The in vitro study objectives were to investigate the effect of arginine (Arg) incorporation in a 5% sodium fluoride (NaF) varnish on its physical and chemical properties including F/Arg release. Six experimental formulations were prepared with L-arginine (L-Arg) and L-arginine monohydrochloride at 2%, 4%, and 8% *w/v* in a 5% NaF varnish, which served as a control. The varnishes were subjected to assessments for adhesion, viscosity, and NaF extraction. Molecular dynamics were simulated to identify post-dynamics total energy for NaF=Arg/Arg>NaF/Arg<NaF concentrations. The Arg/F varnish release profiles were determined in polyacrylic lactate buffer (pH-4.5; 7 days) and artificial saliva (pH-7; 1 h, 24 h, and 12 weeks). Incorporation of L-Arg in NaF varnish significantly influences physical properties ameliorating retention (*p* < 0.001). L-Arg in NaF varnish institutes the Arg-F complex. Molecular dynamics suggests that NaF>Arg concentration denotes the stabilized environment compared to NaF<Arg (*p* < 0.001). The 2% Arg-NaF exhibits periodic perennial Arg/F release and shows significantly higher integrated mean F release than NaF (*p* < 0.001). Incorporating 2% L-arginine in 5% NaF varnish improves its physical properties and renders a stable matrix with enduring higher F/Arg release than control.

## 1. Introduction

Dental caries is a biofilm-dysbiosis triggered chronic disease of dental calcified tissues. The Global Burden of Disease Study in 2016 has estimated that 2.4 billion people worldwide suffer from untreated caries of permanent teeth [1,2]. Globally, 486 million children are reckoned to have caries in their primary teeth [1,2]. Dental caries is caused by persistent fermentable carbohydrates glycolysis and pathogenic biofilm shift leading to cavitation of hard tissues by a biofilm acidic environment [3,4]. To alleviate the global burden of dental caries, strategies impeding biofilm-dysbiosis are indicated to serve as an essential primary preventive regimen.

The long-established evidence on the role of fluorides (F) in caries prevention suggests that non-professional intervention with regular use of fluoridated dentifrices aids in preventing caries, which is dependent on fluoride concentrations [5]. To supplement low levels of F in oral fluids by oral care therapies, professional application of F-containing varnishes are recommended for high caries-risk patients as it adheres to the tooth surface for a prolonged period and serve as a F-releasing reservoir [6]. The reservoir institutes when high concentration F-varnish forms calcium fluoride-like complex precipitation on the enamel surface. The F-containing intervention promotes enamel remineralization and inhibits demineralization while forming acid-resistant fluorapatite. However, F has limited a sustained effect on pathogenic biofilms [7]. Hence, novel interventions that aim at preventing pathogenic biofilm formation are needed to supplement the remineralizing benefits of F-containing interventions.

Arginine (Arg) is a semi-essential amino acid available in micromolar concentrations in saliva. Studies have shown that Arg effectively maintains healthy oral biofilms by improving pH homeostasis through modulation of the oral microbial community [8,9]. The amino acid is selectively metabolized by arginine deiminase system (ADS)-positive commensals (*Streptococcus sanguinis*, *Streptococcus parasanguinis,* and *Streptococcus gordonii)* to ammonia that elevates biofilm pH to counter acidic microenvironments by cariogenic pathogen *Streptococcus mutans* [10]. Additionally, Arg reduces polymicrobial *S. mutans*-containing biofilm biomass and water-insoluble exopolysaccharides production by affecting biofilm-related virulence properties [8,11]. Furthermore, sodium fluoride (NaF) and Arg synergistically have been shown to inhibit *S. mutans* and augment the growth of *S. sanguinis* in a multispecies biofilm [12]. Thus, supplementing Arg to F-containing interventions appears as a promising strategy to prevent biofilm-dysbiosis related to dental caries.

Clinical trials on commercial Arg and F-containing dentifrices have concluded a significant caries-preventive benefit than the control F(alone)-containing dentifrices [13,14]. To date, no study has explored the effect of incorporating Arg in F-containing varnishes on caries-preventive variables like F-release and varnish physical–chemical properties as the interaction of Arg with F might affect theses variables. Therefore, the study objectives were to investigate the effect of Arg incorporation in a 5% sodium fluoride (NaF) varnish on its physical and chemical properties including F/Arg release. The null hypothesis tested in the present study was that Arg incorporation in a 5% NaF varnish has no effect on its physical/chemical properties.

## 2. Materials and Methods

### 2.1. Varnish Preparation and Test Groups

A priori the study design was approved by the Institutional Review Board (IRB) of the University of Hong Kong (Hospital Authority Hong Kong West Cluster Reference #UW 17-544).

A commercially available 5% NaF varnish (Duraphat^®^, Colgate Palmolive Company, New York, NY, USA) was used as a control and for preparation of experimental varnishes. Two variants of Arg were used in the present study—L-arginine (L-Arg) and L-arginine monohydrochloride (L-Arg.HCl) as both the variants have been explored for their caries preventive potential, previously [15,16]. The Arg variants were suspended at 2%, 4%, and 8% *w/v.* in a 10 mL varnish tube dispensed in a sterile container. The suspended Arg was further vigorously mixed with the varnish matrix for 60 sec using a sterile microbrush, which was further used for dispensing the varnish as per the experimental protocol. 

The test groups were as follows:

L-arginine + 5% NaF varnish groups:
  Group 1: 2% L-arginine + 5% NaF varnish (2% Arg-NaF);
  Group 2: 4% L-arginine + 5% NaF varnish (4% Arg-NaF) and;
  Group 3: 8% L-arginine + 5% NaF varnish (8% Arg-NaF).
L-arginine monohydrochloride + 5% NaF varnish:
  Group 4: 2% L-arginine monohydrochloride + 5% NaF varnish (2% Arg.HCl-NaF);
  Group 5: 4% L-arginine monohydrochloride + 5% NaF varnish (4% Arg.HCl-NaF) and;
  Group 6: 8% L-arginine monohydrochloride + 5% NaF varnish (8% Arg.HCl-NaF).
Controls:
  Group 7: 5% NaF varnish (NaF) and;
  Group 8: No varnish.

### 2.2. Physical Characterization 

#### 2.2.1. Varnish Adhesion 

Varnish adhesion and viscosity were assessed using a universal testing machine (ElectroPuls E3000, Instron, Norwood, MA, USA). The adhesion capability of the varnish was determined between 2-stainless steel discs of Φ2.5 cm to obtain pull-off force (in N) using a symmetric pull-off test [17]. The evaluated force was further calculated to obtain the pull-out strength that specifies adhesion using the following formula:

Pull-out strength = (4 × Pull-off Force)/(π × d^2^)

#### 2.2.2. Varnish Viscosity

As the varnish viscosity differed considerably following the incorporation of L-Arg, the dynamic viscosity of the varnish was evaluated with mounted disposable syringe containing varnishes using the universal testing machine (E3000, Instron, Norwood, MA, USA). Displacement velocity of the varnish was set at 1 mm/sec with dislocation at height—1.5 mm over a nozzle area of 0.79 mm^2^ to determine shear rate and shear stress. Viscosity was then computed based on the determined compressive load using the following formula:

Dynamic viscosity (*η_d_*) = Shear stress/shear rate = (Force/Area) × (Height/Velocity)

### 2.3. Chemical Characterization 

#### 2.3.1. Inorganic NaF and Organic F Extraction

After formulation, based on the assumption that incorporating Arg in NaF varnish will lead to the formation of Arg-F that might not be extractable as similar to that of inorganic NaF; NaF extraction or recovery was performed estimating F concentration as in a previous study [18]. To determine the synergistic interaction leading to consequent organic-F genesis, organic-F (i.e., Arg-F precisely per case) estimation was done based on Na-biphenyl reagent reduction of halogenated hydrocarbons to inorganic-F for further analysis using the F electrode. To lead the experiment, chloroform-based primary and secondary NaF extraction were performed; following which the organic-F was reduced with Na-biphenyl complex for inorganic-F estimation [18].

#### 2.3.2. Molecular Interaction of Arginine with Sodium Fluoride

To further explore the molecular interaction between Arg and NaF, molecular mechanics-based dynamics was performed using HyperChem^TM^ Professional v. 8.0.8 (Hypercube Inc., Gainesville, FL, USA). The dynamics was appraised based on different models understanding the concentrations examined in the present study between NaF and Arg using the molecular mechanics framework.

#### 2.3.3. Fluoride and Arginine Release Profiles

The F/Arg release profile of the varnishes was determined in polyacrylic lactate buffer (pH-4.5, 7 days) and artificial saliva (pH-7, 1 h, 24 h, and 12 weeks) using different substrates to identify the release potential in acidic and neutral environments. The acidic environment is to simulate the cariogenic low pH condition. Semicircular polyvinyl strips simulating arches were used to apply varnish for further analysis of F and Arg in lactate buffer as per previous study [19]. The 1 h F/Arg release profile in artificial saliva (Phenol red, 4% NaOH, CaCl_2_, MgCl_2_.H_2_O, KH_2_PO_4_, KCl, HEPES, and NaN_3_ in deionized water) was estimated per varnish applied on 5.5 cm polystyrene petri dish as in a previous study [20]. The 24 h and 12 weeks F/Arg release profile were assessed on the sound human enamel blocks coated with an acid-resistant nail varnish (Revlon^®^, New York, NY, USA) exposing a window of 3 × 3 mm^2^ [21,22]. The substrates were randomly distributed by individual drawing of the specimens per group, until all the specimens were allocated. The substrates for all the experiments were weighed prior and after varnish application. Following each time-point, the substrates with varnish were placed in the respective solutions. The conditions as illustrated per respective studies were followed for the experiments performed in triplicate. The F/Arg estimates were further computed to obtain integrated mean F/Arg release profile per experiment, medium conditions, and time points arithmetically calculated as an average.

#### 2.3.4. Fluoride Analysis

The samples obtained for F analysis were subjected to equal volume of TISAB II (1:1). The F analysis was done using F ion selective electrode (F-ISE; Thermo Fisher Scientific, Waltham, MA, USA) attached to an ion benchtop meter (Orion 2700, Oakton Instruments, Vernon Hills, IL, USA) with an auto-read facility. The F-ISE was calibrated to external standards with 0.1, 1, 10, 100, and 1000 ppm F. To ensure stability, the calibration of the F-ISE was performed before, during, and after the experiments. A new calibration curve was prepared each day with freshly prepared standards. 

The samples were continuously stirred on a magnetic stirrer with micromagnetic bars at 250 rpm room temperature during the analysis. To standardize the electrode orientation, the F-ISE was stationed using a tube holder such that the electrode membrane is in contact with the samples. After each measurement, the electrode was rinsed with deionized water and soaked with dry fibreless laboratory napkins (Kimwipes™ Ex-L, Kimberly-Clark Professional, Roswell, GA, USA) before the next analysis. Care was taken to avoid contact of a napkin with the membrane to prevent electrode damage.

#### 2.3.5. Arginine Detection

Arginine detection was done using the end-point fluorescence spectrophotometric method using a microplate reader (SpectraMax Multimode Microplate Reader, Molecular Devices LLC., San Jose, CA, USA) subjected to serially diluted 8-point freshly prepared Arg standards using 10-ppm L-arginine in deionized water. A working solution of o-phaldialdehyde in 100% ethanol, β-mercaptoethanol, and sodium carbonate was prepared. The working solution and samples were introduced in an opaque-walled fluorescence-based microplate in the ratio of 10:1. The plate was read with excitation—OD_340nm_ and emission—OD_455nm_. A calibration chart was constructed to determined Arg concentrations (in ppm) in the samples obtained per experiment. During the experiment, precautions were taken to keep the working solution away from the light to prevent photointerference. 

### 2.4. Statistical Analysis

All experiments were repeated in at least triplicates. The recorded data were entered in MS Office Excel, which was further subjected to statistical analysis using SPSS v. 25 (IBM Statistics Inc., New York, NY, USA). 

The data on dynamic viscosity, pull-out strength, molecular interaction dynamics, and integrated mean F/Arg release were analyzed by a 1-way ANOVA with Tukey’s HSD post-hoc test. 

The inorganic/organic-F extraction and F/Arg release data were analyzed using a 2-way ANOVA with Bonferroni’s post-hoc test.

Pearson’s correlation coefficient test was applied to identify the statistical relationship between Arg variants/concentrations–dynamic viscosity; Arg variants/concentration–pull out strength; and adhesion–viscosity parameters for Arg variants.

The statistical significance for all the statistical tests was set at α = 0.05.

## 3. Results

### 3.1. Physical Characterization 

**Figure 1:**
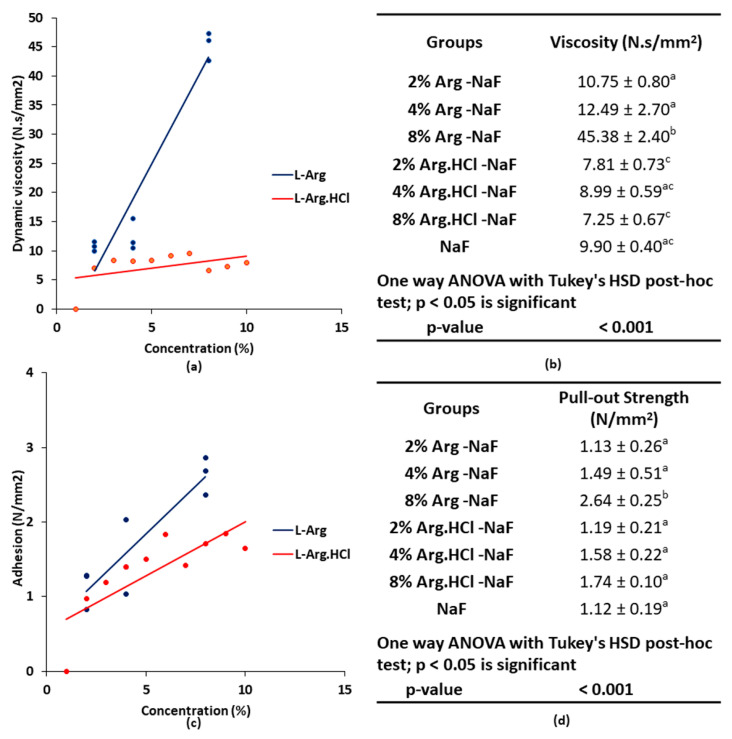
Varnish physical characterization: (**a**) parametric relation between concentration and dynamic viscosity of L-Arg and L-Arg.HCl in NaF varnish; (**b**) the table shows dynamic viscosity of tested varnishes; (**c**) parametric relation between concentration and adhesion of L-Arg and L-Arg.HCl in NaF varnish; and (**d**) the table shows pull-out strength representing adhesion of tested varnishes.

A very strong significant positive correlation coefficient (r = 0.95, *p* < 0.001) was identified between concentration and dynamic viscosity of Arg-NaF varnishes (Figure 1a). The dynamic viscosity of the Arg-NaF varnishes increased with increasing concentrations of Arg. By contrast, the Arg.HCl-NaF varnishes only revealed weak inverse correlation (r = −0.39, *p* = 0.298; Figure 1a). The viscosity of the 8% Arg-NaF varnish was significantly higher than the other tested varnishes (*p* < 0.001; Figure 1b). Likewise, a very strong positive significant correlation coefficient (r = 0.91, *p* = 0.001) was observed between pull-out strength and concentration of the Arg-NaF varnishes (Figure 1c). Similar to viscosity, the pull-out strength for the 8% Arg-NaF varnish was significantly higher than the other tested varnishes (*p* < 0.001; Figure 1d). The physical characterization of the Arg-NaF varnishes showed that incorporating Arg in NaF varnish increased its viscosity and promoted retention, which is beneficial for clinical application on the enamel surface.

### 3.2. Chemical Characterization 

#### 3.2.1. Inorganic NaF and Organic F Extraction

**Figure 2:**
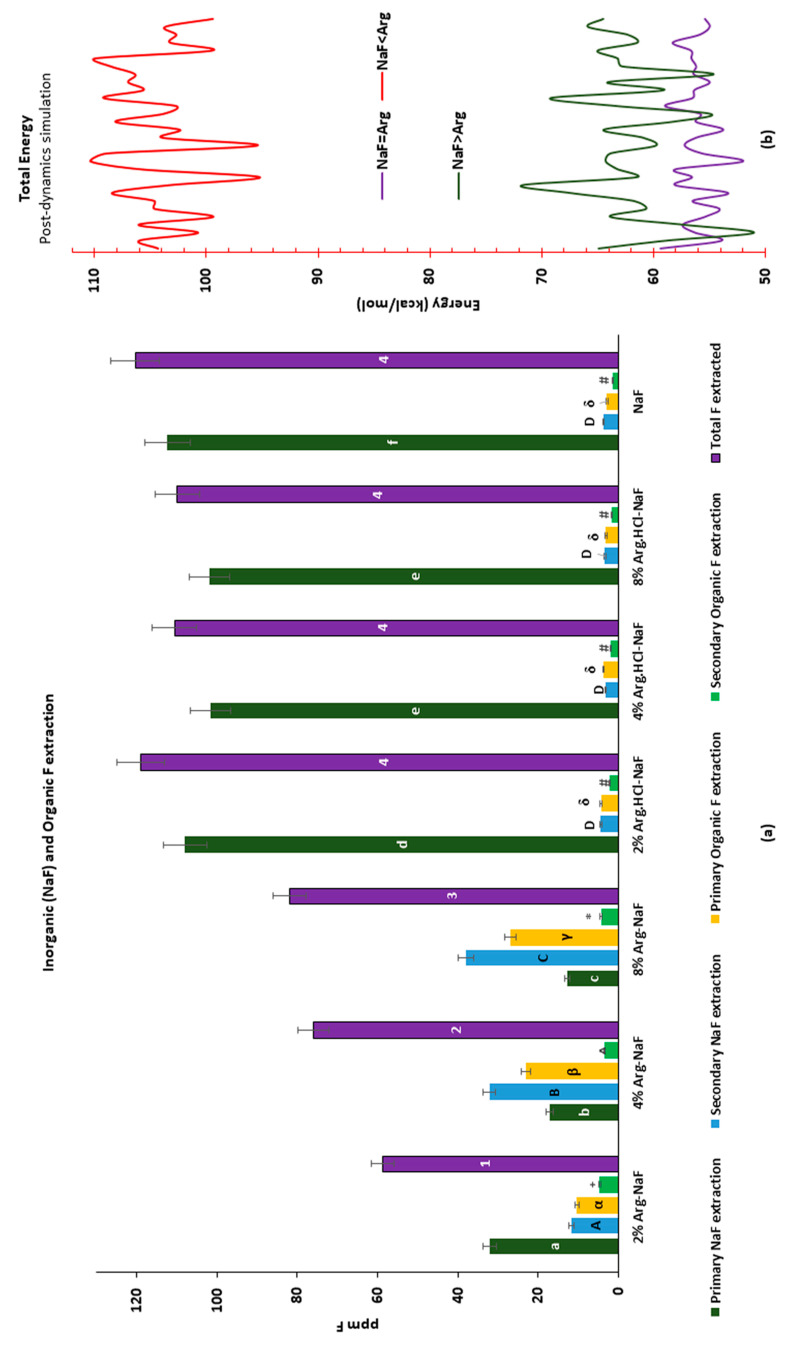
Varnish chemical characterization: (**a**) inorganic (NaF) and organic F extraction/recovery. (**b**) Molecular dynamics: total energy post-molecular dynamics using molecular mechanics setup for 1.000ps@0.001 steps with simulation temperature: 300 K for models with concentrations: NaF = Arg, NaF > Arg, and NaF < Arg.

For the Arg-NaF varnishes, the mean primary NaF extraction decreased with increasing Arg concentration with the lowest in the 8% Arg-NaF varnish (*p* < 0.05; Figure 2a). However, the declining order was reversed while secondary NaF extraction was attempted (*p* < 0.05). In contrast, no significant difference in mean secondary NaF extraction was observed between the Arg.HCl-NaF and NaF varnishes (*p* > 0.05). The results showed that L-Arg in NaF varnish exhibited some concentration-dependent Arg-F interaction.

Conversely, the primary organic-F (Arg-F) extraction from the Arg-NaF varnishes increased with increasing concentrations of Arg (*p* < 0.05); while no significant difference was discerned between the Arg.HCl-NaF and NaF varnishes (*p* > 0.05). The secondary organic-F extraction from the 2% Arg-NaF varnish was significantly higher than the other varnishes (*p* < 0.05). The total F extracted from the Arg-NaF varnishes was significantly lower than the Arg.HCl-NaF and NaF varnishes (*p* < 0.05), suggesting a dose-dependent chemical interaction between L-Arg and NaF (Figure 2a).

#### 3.2.2. Molecular Interaction of Arginine with Sodium Fluoride

Figure 2b shows the dynamics between the molecules NaF and Arg in different models, i.e., NaF > Arg, NaF < Arg, and NaF = Arg using molecular mechanics configuration estimating energy levels in kcal/mol against simulated temperature based on molecular dynamics computations. The time-domain energy estimated at 1 ps for NaF > Arg and NaF = Arg were significantly lower than NaF < Arg (*p* < 0.001; Figure 2b). Although, the energy level for NaF > Arg was higher than NaF = Arg (*p* < 0.05), a few energy overlays were obvious as shown in Figure 2b. The simulations emphasize that NaF < Arg with time-domain molecular dynamics based on mechanics (MM+) framework suggested higher-order energy levels; whereas NaF = Arg and NaF > Arg indicated comparable stability. Thus, NaF > Arg, similar to control NaF = Arg, denoted stabilized environs. 

#### 3.2.3. Fluoride and Arg Release Profile 

The weight of varnish applied on substrates subjected to different experimental conditions validated no significant difference between the tested varnishes and the control NaF varnish (*p* > 0.05; Table 1).

##### Fluoride Release in Polyacrylic Lactate Buffer and Artificial Saliva

**Figure 3:**
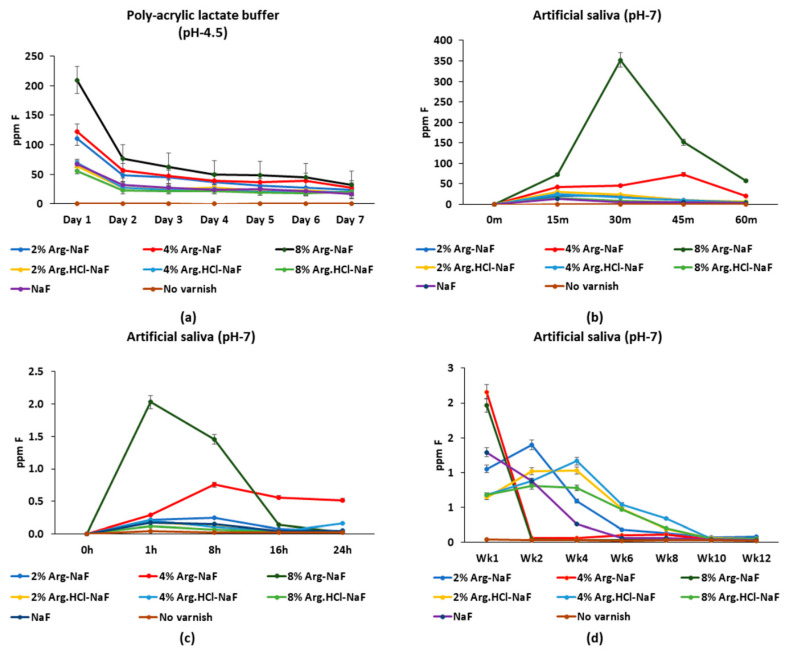
Mean fluoride release profiles of tested varnishes applied on: (**a**) polyvinyl strips to assess release in polyacrylic lactate buffer over 7 days; (**b**) 5.5 cm petri-dish to assess release in artificial saliva over 60 min; (**c**) facial surface of quadri-sectioned tooth with exposed window (3 × 3 mm^2^) to assess release in artificial saliva over 24 h; and (**d**) facial surface of quadri-sectioned tooth with exposed window (3 × 3 mm^2^) to assess release in artificial saliva over 12 weeks.

Fluoride release from the Arg-NaF varnishes in polyacrylic lactate buffer was significantly higher than the Arg.HCl-NaF and the control NaF varnishes at day 1 (*p* < 0.001; Figure 3a). Fluoride release from the 8% Arg-NaF varnish in artificial saliva was significantly higher than the other varnishes until 45 min (*p* < 0.05; Figure 3b), which continued to be higher than the other tested varnishes between 1 and 8 h (*p* < 0.05) with a rapid decline at 16 h, to a level similar to other varnishes (*p* > 0.05; Figure 3c). At week 1, F release from the 4% and 8% Arg-NaF varnishes was significantly higher than the other varnishes (*p* < 0.05), which attenuated considerably at week 2 (Figure 3d). However, at week 2, the 2% Arg-NaF varnish showed significantly higher F release than the other tested varnishes and continued to have higher F release than the NaF varnish until week 4 (*p* < 0.05). The F release profile of the 4% and 8% Arg-NaF varnishes was not perpetual, with limiting long-lasting effects. In contrast, F release from the 2% Arg-NaF varnish was perennial and significantly higher than the NaF varnish (*p* < 0.05).

##### Arginine Release in Polyacrylic Lactate Buffer and Artificial Saliva

**Figure 4:**
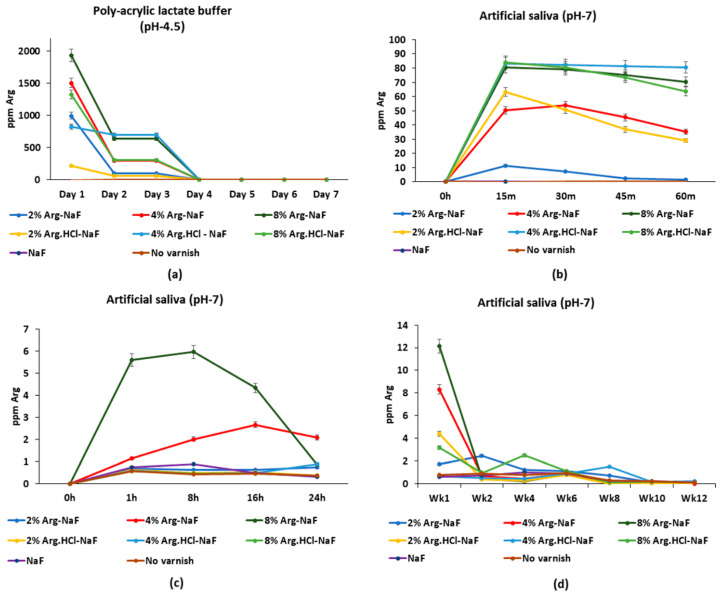
Mean arginine release profiles of tested varnishes applied on: (**a**) polyvinyl strips to assess release in polyacrylic lactate buffer over 7 days; (**b**) 5.5 cm petri-dish to assess release in artificial saliva over 60 min; (**c**) facial surface of quadri-sectioned tooth with exposed window (3 × 3 mm^2^) to assess release in artificial saliva over 24 h; and (**d**) facial surface of quadri-sectioned tooth with exposed window (3 × 3 mm^2^) to assess release in artificial saliva over 12 weeks.

The 8% Arg-NaF varnish exhibited significantly higher Arg release in polyacrylic lactate buffer than the other varnishes at day 1 (*p* < 0.05), which significantly decreased (*p* < 0.001) to a level similar to the other varnishes at day 4 (*p* > 0.05; Figure 4a). Arginine release from the 8% Arg-NaF, 4% Arg.HCl-NaF, and 8% Arg.HCl-NaF varnishes in artificial saliva was significantly higher than the other varnishes until 45 min (*p* > 0.05; Figure 4b). In general, Arg release from the 4% and 8% Arg-NaF varnishes was higher than the other varnishes from 8 to 16 h (Figure 4c). The 8% Arg-NaF varnish continued to release significantly more Arg at week 1 than the other varnishes (*p* < 0.05), whereas the 2% Arg-NaF varnish released significantly higher Arg at week 2 when compared to the other varnishes (*p* < 0.05; Figure 4d). Arginine release from the 2% Arg-NaF varnish was more consistent when compared to the other varnishes, which dropped significantly at week 10 (*p* < 0.001). Arginine release profile for the 4% and 8% Arg-NaF varnishes was sporadic; while the 2% Arg-NaF varnish was more enduring. Hence, the 2% Arg-NaF varnish exhibited perennial Arg and F release.

##### Integrated Fluoride/Arginine Release 

**Figure 5:**
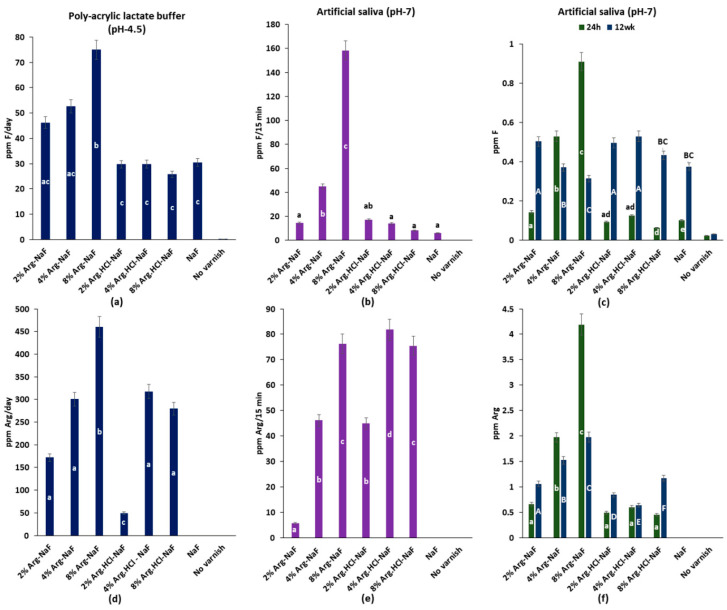
Integrated mean release of (**a**) fluoride in polyacrylic lactate buffer over 7 days; (**b**) fluoride in artificial saliva over 60 min; (**c**) fluoride in artificial saliva over 24 h and 12 weeks; (**d**) arginine in polyacrylic lactate buffer over 7 days; (**e**) arginine in artificial saliva over 60 min; and (**f**) arginine in artificial saliva over 24 h and 12 weeks.

The 8% Arg-NaF varnish showed significantly higher integrated mean F release than the other varnishes in polyacrylic lactate buffer (for 7 days), 60 min, and 24 h in artificial saliva (*p* < 0.05) (Figure 5a–c). However, the integrated mean F release in saliva over 12 weeks for 2% Arg-NaF, 2% Arg.HCl-NaF, and 4% Arg.HCl-NaF varnishes was significantly higher than the other varnishes (*p* < 0.05; Figure 5c). The integrated mean Arg release for the 8% Arg-NaF varnish in polyacrylic lactate buffer was significantly higher than the other varnishes (*p* < 0.05; Figure 5d). Conversely, the integrated mean Arg release for the 4% Arg.HCl-NaF varnish was significantly higher than the other varnishes in artificial saliva for 60 min (*p* < 0.05; Figure 5e). The 8% Arg-NaF varnish exhibited the highest integrated mean Arg release in artificial saliva (24 h/12 weeks) when compared to the other varnishes (*p* < 0.05; Figure 5f). 

The integrated mean F/Arg release profile showed that the 2% Arg-NaF varnish presented with a higher F release than NaF varnish over a prolonged period of 12 weeks (*p* < 0.05), whereas the 8% Arg-NaF varnish showed a higher Arg release throughout, irrespective of the pH, environment, and duration.

## 4. Discussion

The present study examined the effect of Arg incorporation in a 5% NaF varnish on its physical–chemical properties and discerned that incorporating L-Arg in NaF varnish affected the variables like varnish adhesion, viscosity, inorganic F content, and F release with a simultaneous potential to release Arg from the varnish matrix. Conversely, the incorporation of L-Arg.HCl in NaF varnish has no significant effect on the varnish physical and chemical properties. Thus, based on the results of the study, the null hypothesis that Arg incorporation in a 5% NaF varnish had no effect on its physical/chemical properties had to be partially rejected.

Previous clinical studies have reported the promising role of Arg in caries prevention [13,23]. Thus, incorporating Arg in F-containing varnish appears to be a good strategy for establishing oral ecological homeostasis and preventing microbial dysbiosis contributory to the development of dental caries. As an enduring preventive measure, Arg-F varnish (2% Arg-NaF) aids to target both the pathogenic biofilms and the remineralization–demineralization dynamics of teeth [24,25] by its prolonged retention on the tooth surface. Therefore, the Arg-F varnish counters the long-existing limitations of F with enhanced physical–chemical properties of the current 5% NaF varnish formulations.

The viscosities of the Arg-F varnish differed considerably when compared to the control NaF varnish per concentration increase of L-Arg. The 8% Arg-NaF demonstrated the highest viscosity; whereas the viscosity with Arg.HCl-NaF was not affected, which might be due to the limited/non-interaction of Arg.HCl with the components of NaF varnish. It is postulated that L-Arg might act as a filler for NaF, which positively affects the viscosity of NaF varnish, increasing its retention on tooth surface. Further chemical evaluation is necessary to identify whether the change in physical properties was due to the interaction dynamics or its physical annexure to the varnish components.

The molecular dynamics also identified that the reactivity of the interactive elements was based on the energy dependent phase, which would need further investigations. However, it was quite lucid that Arg in lower concentrations than NaF would provide a stabilized environment for functionalization when compared to Arg at higher concentrations. All the data showed that the incorporation of 2% L-Arg in a 5% NaF varnish exhibited synergism, enhancing its physical–chemical properties with stabilized matrix.

The inorganic–organic F extraction for Arg-NaF varnishes affirmed a concentration-dependent trend with each extraction level; whereas little but insignificant change was evident in Arg.HCl-NaF varnishes compared to NaF varnish. While primary NaF extraction was the least with the 8% Arg-NaF varnish, the secondary NaF extraction was significantly higher than the 2% and 4% Arg-NaF varnishes, similar to the primary organic F extraction with Arg-NaF varnishes. This could be due to the reaction between the insoluble NaF in varnish with L-Arg forming the Arg-F complex; whereas the soluble NaF content remains intact with release. The Arg-F formed further increases its solubility during subsequent media exposure, being organic thereby increasing reactivity with the enamel surface over time as seen with NaF varnish [26].

The formation of Arg-F complex-containing NaF may also lead to the delay in NaF extraction due to the trapping of NaF intermediary immixture, which was eventually released in the aqueous segment. Although little, significant higher secondary organic-F was extracted from the 2% Arg-NaF varnish than the other groups, suggestive of a stable matrix with enmeshed organic-F despite the reduction dynamism. Overall, the analysis identified that the incorporation of Arg in NaF varnish significantly influenced its physical–chemical properties; with 2% Arg-NaF being a constructive variable of Arg-F complex that enhanced functional potential of NaF varnish.

The 8% Arg-NaF varnish demonstrated a significantly higher initial Arg and F release than the other groups. The F release profile of the 8% Arg-NaF varnish contradicted the known concept that low-viscosity resins favored F diffusion [21], which rejected the explanation for the results of molecular dynamics with Arg > NaF concentrations. Hence, the modified physical properties of the Arg-NaF varnishes did not influence the F and Arg release profiles of the tested varnishes, rather it was the chemical interplay that led to the present results.

Arginine alone is capable of maintaining the alkaline pH that favors the growth of alkalogenic commensals and makes the environment less conducive for the growth of acidogenic/aciduric bacteria. However, with higher concentrations of L-Arg (i.e., 8% *w/v*) a higher order pH shift might pose concern of biofilm over alkalization and possibly supporting the growth of periodontal pathogens—*Porphyromonas gingivalis* [12]. Hence, one needs to be cautious with higher L-Arg concentrations as it might do more harm than benefit to the microflora. Additionally, it is known that with a decreasing pH environment, there is an increase in F reactivity with enamel [27]. However, studies have shown in the past that Arg improves F uptake in enamel incipient caries-like lesion [28]. Therefore, there could be a potential for Arg to increase F uptake given that its presence is independent of environmental pH.

The F release potential of 5% NaF varnish has been examined by several studies [18,29], which demonstrated that the release potential is steady and stable. However, with the incorporation of L-arginine, the dynamics of F release changed with higher concentrations (4% and 8%) demonstrating higher F release. Additionally, previous studies incorporated different compounds (Na-trimetaphosphate, Ca-glycerophosphate, titanium-F, etc.) in NaF varnish and examined its F release potential [30,31,32]. With higher F release, the remineralization effect was not enhanced. Thus, a stable matrix with durable F release for a prolonged period is desirable, which was quite evident in the 2% Arg-NaF group.

The present study comprehensively exhibited the potential of L-arginine to affect changes in the physical and chemical properties of a 5% NaF varnish. The study limits itself to investigate the varnish properties while the effect of a potential Arg-F varnish combination (2% Arg-NaF) for caries prevention still needs to be discerned on dental hard tissues like enamel. Therefore, future studies are needed to examine the caries-preventive effect of the assessed combinations (in the present study) on artificial incipient enamel caries-like lesions demonstrating the remineralization potential of the interventions. Following which, studies are needed to identify the cytotoxic effects of the combinations as an unstable matrix with higher F release might be contributory to toxicity.

## 5. Conclusions

Under the conditions of the present study, we conclude that:

1. L-arginine in 5% NaF varnish affected varnish physical and chemical properties; while L-arginine monohydrochloride in 5% NaF varnish had a non-contributory effect on varnish properties.
2. Incorporating 2% L-arginine in 5% NaF varnish improved its physical properties and renders a stable matrix with enduring higher F/Arg release than control.

## Figures and Tables

**Table 1:** Varnish weight on substrates.

Varnish Weight on Substrates (g)				
Groups	7-Day Demineralization Buffer	1 h Artificial Saliva	24 h Artificial Saliva	12 w Artificial Saliva
**2% Arg -NaF**	0.67 ± 0.08 ^a^	0.20 ± 0.00	0.003 ± 0.001	0.003 ± 0.001
**4% Arg -NaF**	0.77 ± 0.20 ^a^	0.20 ± 0.00	0.003 ± 0.001	0.003 ± 0.001
**8% Arg -NaF**	0.72 ± 0.14 ^a^	0.20 ± 0.00	0.003 ± 0.001	0.003 ± 0.001
**2% Arg.HCl -NaF**	0.69 ± 0.17 ^a^	0.20 ± 0.00	0.003 ± 0.001	0.003 ± 0.001
**4% Arg.HCl -NaF**	0.79 ± 0.10 ^a^	0.20 ± 0.00	0.003 ± 0.001	0.003 ± 0.001
**8% Arg.HCl -NaF**	0.59 ± 0.08 ^a^	0.20 ± 0.00	0.003 ± 0.001	0.003 ± 0.001
**NaF**	0.70 ± 0.13 ^a^	0.20 ± 0.00	0.003 ± 0.001	0.003 ± 0.001
**No varnish**	0.05 ± 0.00 ^b^	-	-	-
*One-way ANOVA with Tukey’s HSD post-hoc test; p < 0.05 is significant. Different superscripts identify significant differences between test groups*				
*p*-value	<0.001	0.174	0.654	0.490
